# A hemoglobin-based oxygen carrier sensitized Cisplatin based chemotherapy in hepatocellular carcinoma

**DOI:** 10.18632/oncotarget.19672

**Published:** 2017-07-28

**Authors:** Xiang Qi, Bing L. Wong, Sze Hang Lau, Kevin Tak-Pan Ng, Sui Yi Kwok, Chris Kin-Wai Sun, Fei Chuen Tzang, Yan Shao, Chang Xian Li, Wei Geng, Chang Chun Ling, Yuen Yuen Ma, Xiao Bing Liu, Hui Liu, Jiang Liu, Wai Ho Yeung, Chung Mau Lo, Kwan Man

**Affiliations:** ^1^ Department of Surgery, The University of Hong Kong, Hong Kong, China; ^2^ Collaborative Innovation Center for Diagnosis and Treatment of Infectious Diseases, Hangzhou, China; ^3^ New β Innovation Limited, 18/F Chevalier Commercial Centre, Hong Kong, China

**Keywords:** hemoglobin-based oxygen carrier, chemoresistance, HCC, Cisplatin, intravital imaging

## Abstract

**Background and Objective:**

Our previous study showed that liver graft injury not only promotes tumor recurrence, but also induces chemoresistance in recurrent HCC after liver transplantation. Recently, we found that the hemoglobin-based oxygen carrier“YQ23” significantly ameliorates hepatic IR injury and prevent tumor recurrence. Here, we intended to explore the novel therapeutic strategy using oxygen carrier “YQ23”to sensitize chemotherapy in HCC.

**Methods:**

To investigate the role of YQ23 combined with Cisplatin, the proliferation of HCC cells was examined under combined treatment by MTT and colony formation. To explore the effect of YQ23 on sensitization of Cisplatin based chemotherapy, the orthotopic liver cancer model was established. To characterize the delivery of YQ23 in tumor tissue, the intravital imaging system was applied for longitudinal observation in ectopic liver cancer model. The distribution of YQ23 was examined by IVIS spectrum.

**Results:**

YQ23 significantly suppressed the proliferation of HCC cells under Cisplatin treatment in a dose and time dependent manner. Moreover, YQ23 administration significantly sensitized Cisplatin based chemotherapy in orthotopic liver cancer model. Down-regulation of DHFR may be one of the reasons for YQ23 sensitizing Cisplatin based chemotherapy. Real-time intravital imaging showed that YQ23 accumulated in the tumor tissue and maintained as long as 3 days in ectopic liver cancer model. The IVIS spectrum examination showed that YQ23 distributed mainly at liver and bladder within the first 36 hours after administration in orthotopic liver cancer model.

**Conclusion:**

YQ23 treatment may be a potential therapeutic strategy to sensitize chemotherapy in HCC.

## INTRODUCTION

Hepatocellular carcinoma (HCC) is the sixth most common cancer and ranks as high as third for cancer-related deaths worldwide [1]. Liver transplantation is the effective treatment for selected HCC patients. Our previous study showed that severe hepatic ischemia-reperfusion (IR) injury not only promotes tumor recurrence, but also induces chemoresistance in recurrent HCC after liver transplantation [2]. Recently, we found that the hemoglobin-based oxygen carrier“YQ23” significantly ameliorates hepatic IR injury and prevent tumor recurrence [3]. Therefore, investigation of the role of YQ23 in sensitization of chemotherapy in HCC may help for developing novel therapeutic strategy targeting at HCC resistance after liver transplantation.

Hypoxia is one of the major reasons to induce chemoresistance of HCC cells [4]. It has been reported that hypoxia could promote chemoresistance in HCC through hypoxia induced multidrug-resistance related gene, such as multidrug resistance transporter P-glycoprotein [5], MDR1 (Multi-Drug Resistance Gene 1), MRP1 (Multidrug Resistance-associated Protein 1) and LRP (Lung Resistance-related Protein) [6]. Hypoxia protects HCC cells from chemotherapy-induced apoptosis [7]. Moreover, HIF-1α (Hypoxia-Inducible Factor 1-alpha) promotes hypoxia induced chemoresistance in HCC through up-regulation of WSB-1 (WD repeat and SOCS Box-containing protein 1) [8]. Nuclear translocation and activation of YAP (Yes-Associated Protein 1) also contributes to hypoxia induced chemoresistance in HCC [9]. Recent reports showed that Egr-1 (Early growth response protein 1) promotes chemoresistance in HCC through hypoxia induced autophagy [10]. As hypoxia is inevitable in liver transplantation and hypoxia induced chemoresistance could occur in recurrent HCC post-transplantation [2], attenuation of hypoxia environment of tumor cells using oxygen carrier may provide new insight for developing therapeutic strategy targeting at chemoresistance in HCC, especially for recurrent HCC after liver transplantation.

YQ23 product is the stabilized non-polymeric cross-linked tetrameric hemoglobin (65 kDa) with undetectable/low level of dimeric hemoglobin (32 kDa), phospholipid, DNA impurities and protein impurities. The hemoglobin-based oxygen carrier was originally developed as an alternative strategy for erythrocytes transfusion [11, 12]. It has been reported that the artificial oxygen carrier can effectively improve oxygenation in liver, kidney and other organs [3, 13, 14]. Our previous study showed that the hemoglobin-based oxygen carrier“YQ23” not only ameliorates hepatic ischemia-reperfusion (IR) injury, but also effectively reduced the incidence of tumor recurrence [3]. Our recent study showed that hepatic IR injury not only promotes tumor recurrence, but also induces chemoresistance in recurrent HCC after liver transplantation [2]. The role of YQ23 in sensitization of chemotherapy in HCC remains to be further investigated.

With the development of imaging technology, the modality of molecular intravital imaging currently enables us to directly see the tumor cells and molecules interaction in a live animal. Optical technique of functional imaging has well developed in clinical situation, including magnetic resonance imaging (MRI), positron emission tomography (PET) or computed tomography (CT). However, the major challenge in such technique is the limited penetration depth imposed by tissue turbidity, which avoids the possibility of utilizing high resolution microscope [15]. Here, we utilized a novel intravital imaging system using dorsal window chamber which could overcome such limitation. The intravital imaging of dorsal window chamber has been already applied in detection of vascularization with the advantages of longitudinal observation and high resolution in animal model [16]. The establishment of new platform of imaging would allow us to kinetically observe the drug delivery within tumor tissues with high resolution in a live animal.

In the present study, we aimed to explore the role of hemoglobin-based oxygen carrier “YQ23” in sensitization of Cisplatin based chemotherapy in HCC. We firstly studied the role of YQ23 in proliferation of HCC under the presence of Cisplatin using MTT and colony formation assay. Then, we examined the effect of YQ23 treatment combined with Cisplatin on tumor growth in orthotopic liver cancer model and further explore the underlying mechanism. After that, we investigated the delivery and distribution of YQ23 using real-time intravital imaging system. We hoped that our study could provide the evidences for using oxygen carrier YQ23 to sensitize Cisplatin based chemotherapy of HCC after liver transplantation.

## RESULTS

### YQ23 significantly suppressed the proliferation of HCC cells under Cisplatin treatment in a dose and time dependent manner

**Figure 1:**
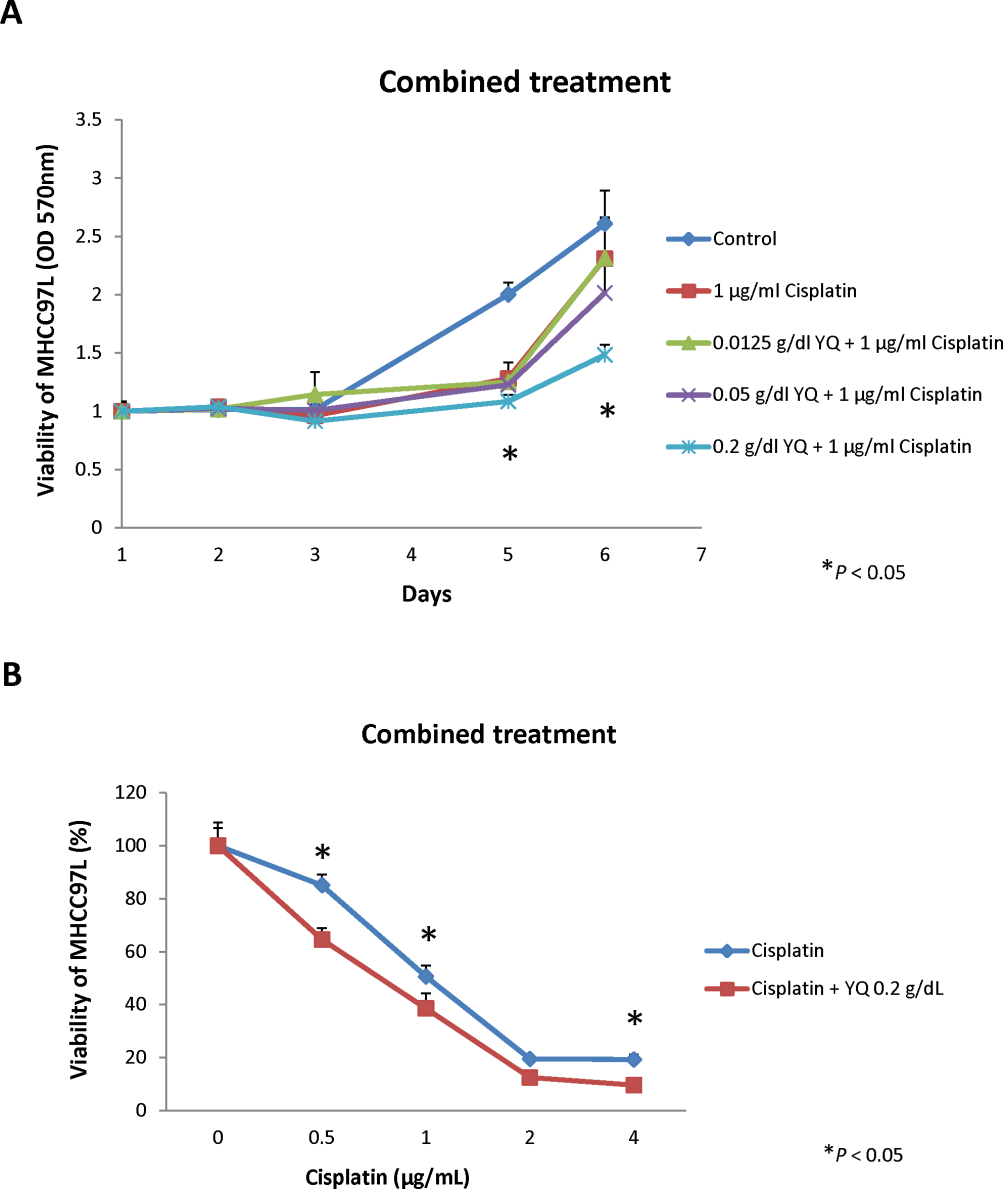
Oxygen carrier YQ23 significantly sensitized Cisplatin treatment by MTT assay *in vitro* **(A)** YQ23 administration significantly suppressed the proliferation of HCC cells under Cisplatin treatment in a dose dependent manner. **(B)** YQ23 could most significantly sensitize Cisplatin treatment at lower dosage of Cisplatin at day 7.

**Figure 2:**
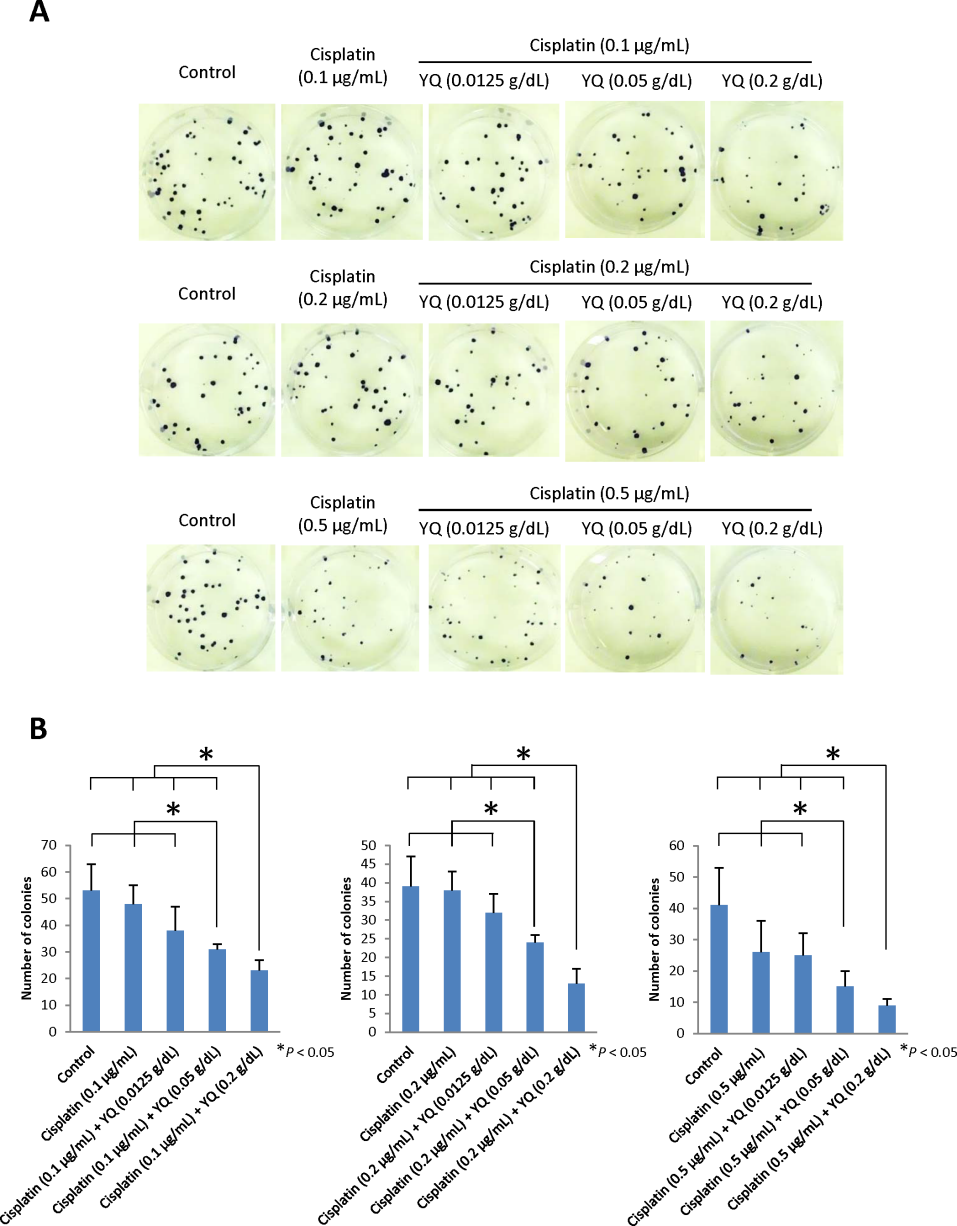
Oxygen carrier YQ23 significantly suppressed the colony formation of HCC cells under Cisplatin in a dose dependent manner **(A)** Images of colony formation for 3 weeks. **(B)** Quantification and statistical analysis for colony formation.

To examine whether YQ23 may sensitize Cisplatin based chemotherapy in HCC, MHCC97L cells were cultured under the presence of Cisplatin (1μg/mL) and treated with different dosage of YQ23. The dosage of Cisplatin was determined by dose-response curve. We found that YQ23 significantly suppressed the proliferation of HCC cells under Cisplatin treatment in a dose dependent manner by MTT assay (Figure 1A). The peak effect of YQ23 was shown at the concentration of 0.2g/dL. YQ23 significantly suppressed the proliferation of tumor cells at day 5 and 6. It implied that the suppressive effect of YQ23 was more obvious at later phase. No significant effect was observed by YQ23 single treatment (Supplementary Figure 1). After that, we further examine the suppressive effect of YQ23 (0.2g/dL) under different concentration of Cisplatin (Figure 1B). We found that YQ23 could most significantly sensitize Cisplatin based chemotherapy at lower dosage of Cisplatin (0.5μg/mL) at day 7 (Figure 1B). In order to examine the long time effect of YQ23 on tumor cells growth, MHCC97L cells were cultured under the combined treatment for 2 and 3 weeks. Again, we observed that YQ23 significantly suppressed the proliferation of HCC cells under Cisplatin in a dose dependent manner in colony formation assay (Figure 2A and 2B).

### YQ23 significantly sensitized Cisplatin based chemotherapy in orthotopic xenograft liver cancer model

**Figure 3:**
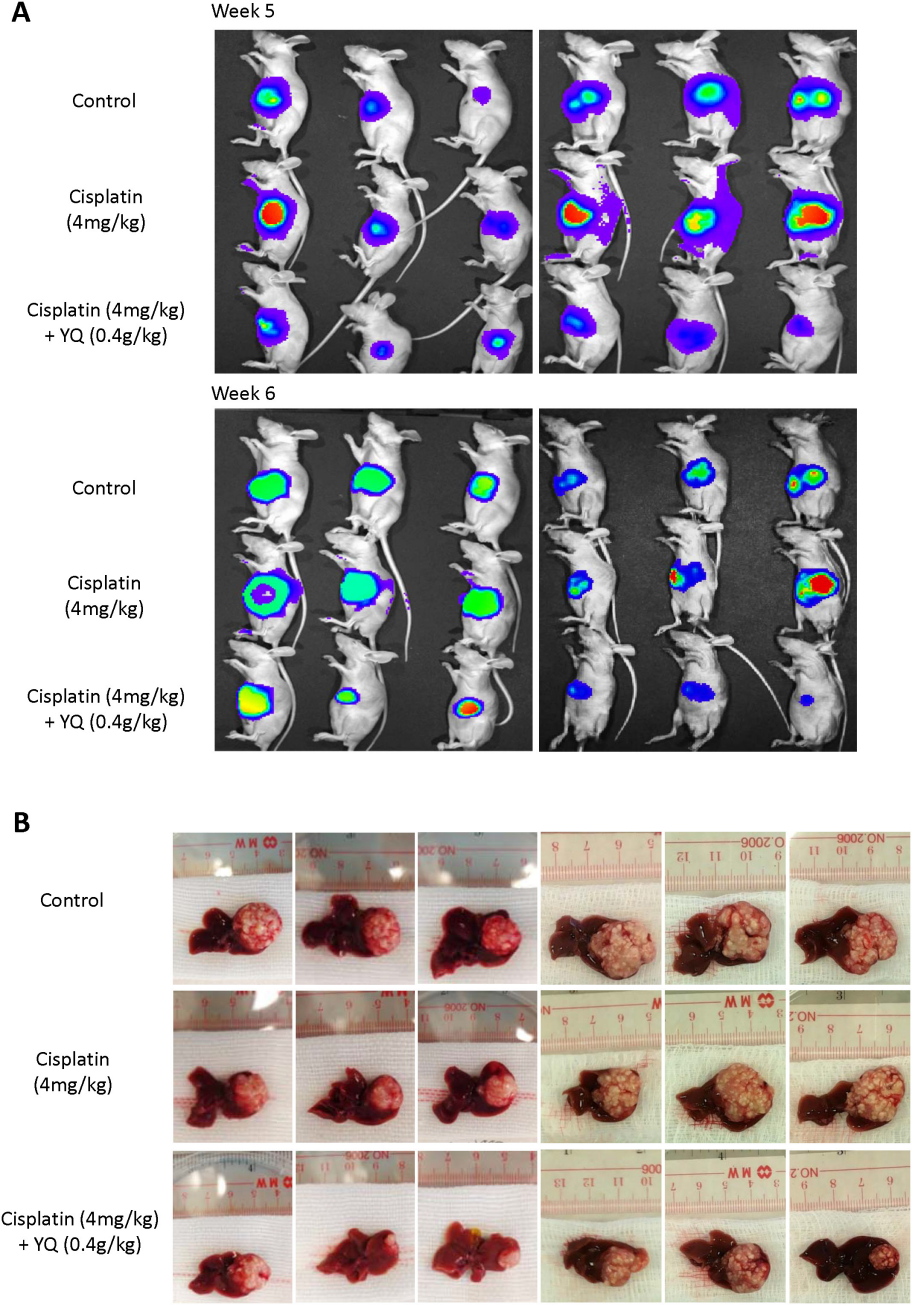
Oxygen carrier YQ23 significantly sensitized Cisplatin treatment *in vivo* **(A)** YQ23 administration kinetically sensitized Cisplatin based chemotherapy in orthotopic xenograft liver cancer model. **(B)** The tumor volume was significantly lower by YQ23 treatment combined with Cisplatin therapy when the nude mice were sacrificed.

**Figure 4:**
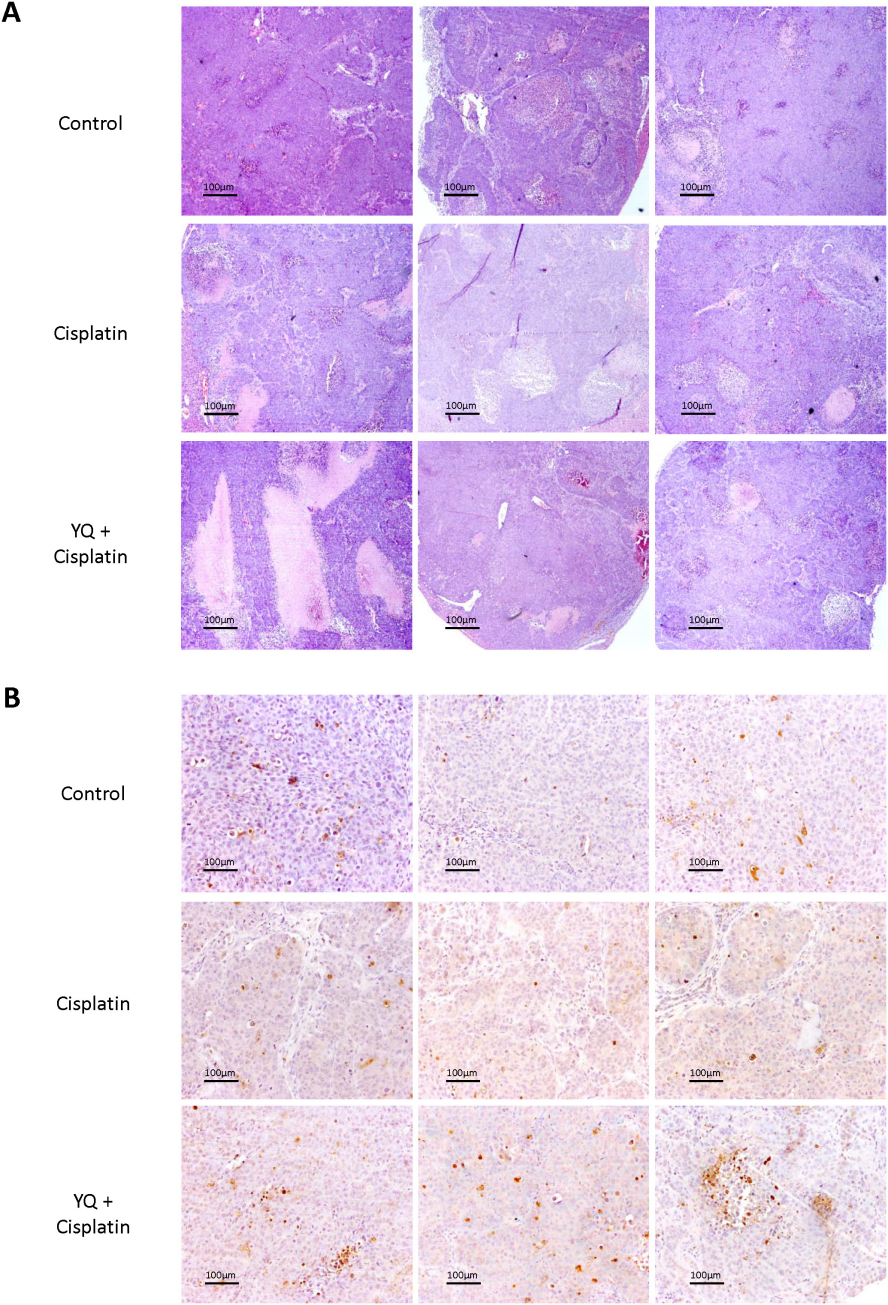
Oxygen carrier YQ23 combined with Cisplatin treatment induced more necrosis and apoptosis in tumor tissues **(A)** More necrotic areas were observed in combined treatment group by H&E staining (400×). **(B)** More apoptotic cells were induced by combined treatment examined by TUNEL assay (400×).

To examine whether YQ23 may sensitize Cisplatin based chemotherapy *in vivo*, the nude mice orthotopic xenograft liver cancer model was established. Cisplatin and YQ23 were administrated into the mice once a week after tumor implantation. The experiments were performed in duplicate. We observed that YQ23 kinetically sensitized Cisplatin based chemotherapy at week 5 and week 6 after tumor implantation (Figure 3A). No significant effect was observed by Cisplatin single treatment. The tumor volume was significantly lower by YQ23 treatment combined with Cisplatin therapy when the nude mice were sacrificed (Figure 3B). More necrotic areas were observed in combined treatment group by H&E (Hematoxylin and eosin) staining (Figure 4A). More apoptotic cells were induced by combined treatment examined by TUNEL (Terminal deoxynucleotidyl transferase dUTP nick end labeling) assay (Figure 4B).

### Down-regulation of dihydrofolate reductase (DHFR) may be one of the reasons for YQ23 sensitizing Cisplatin based chemotherapy

**Figure 5:**
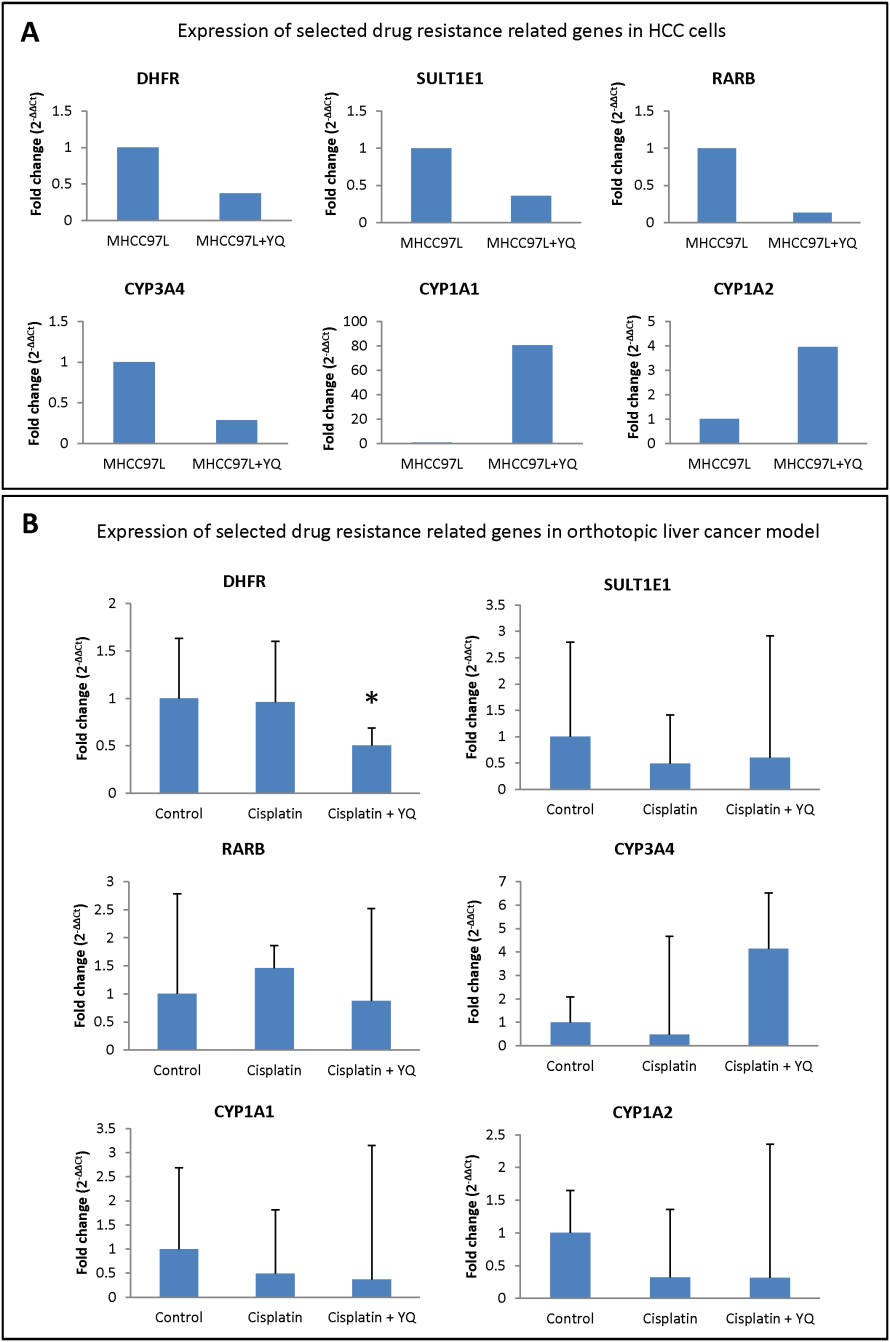
Down-regulation of dihydrofolate reductase (DHFR) may be one of the reasons for YQ23 sensitizing Cisplatin based chemotherapy **(A)** RT^2^ Profiler PCR array showed that 4 down-regulated genes (DHFR, SULT1E1, RARB and CYP3A4) and 2 up-regulated genes (CYP1A1 and CYP1A2) were identified in HCC cells after YQ23 treatment. **(B)** Only DHFR was significantly down-regulated upon YQ23 treatment combined with Cisplatin in orthotopic xenograft liver cancer model. *P<0.05.

In order to explore the mechanism of YQ23 sensitizing Cisplatin based chemotherapy, the drug resistance related pathways were further analyzed using RT^2^ Profiler PCR array. Among 84 drug resistance related genes, using 2-fold as cutoff point, 4 down-regulated genes (DHFR, SULT1E1, RARB and CYP3A4) and 2 up-regulated genes (CYP1A1 and CYP1A2) were identified in HCC cells after YQ23 treatment (Table 1, Figure 5A). After that, the expressions of these 6 potential gene candidates were further validated in orthotopic xenograft liver cancer model. We found that only DHFR was significantly down-regulated upon YQ23 treatment combined with Cisplatin (Figure 5B). No significant change was found for the other 5 potential candidates. It implied that down-regulation of DHRF may be one of the reasons responsible for YQ23 sensitizing Cisplatin based chemotherapy.

**Table 1:** Differentially expressed drug resistance related genes in HCC cells after YQ treatment

Symbol	GenBank	UniGene	Description
Down-regulated			
DHFR	NM_000791	Hs.592364	Dihydrofolate reductase
SULT1E1	NM_005420	Hs.479898	Sulfotransferase family 1E, estrogen-preferring, member 1
RARB	NM_000965	Hs.654490	Retinoic acid receptor, beta
CYP3A4	NM_017460	Hs.654391	Cytochrome P450, family 3, subfamily A, polypeptide 4
Up-regulated			
CYP1A1	NM_000499	Hs.72912	Cytochrome P450, family 1, subfamily A, polypeptide 1
CYP1A2	NM_000761	Hs.1361	Cytochrome P450, family 1, subfamily A, polypeptide 2

The 84 drug resistance related genes were involved in the PCR array. The genes encoding important enzymes for drug resistance (such as the P-glycoproteins), phase I metabolism (specifically the P450 family), and phase II metabolism (such as various covalent modification enzymes) were all represented on the array. Cancer-related genes involved in aspects of resistance are also included on the array such as DNA repair enzymes, cell cycle regulators, growth factor and hormone receptors and transcription factors.

### Real-time intravital imaging (Confocal) showed that YQ23 accumulated in the tumor tissue in ectopic xenograft liver cancer model using dorsal window chamber

**Figure 6:**
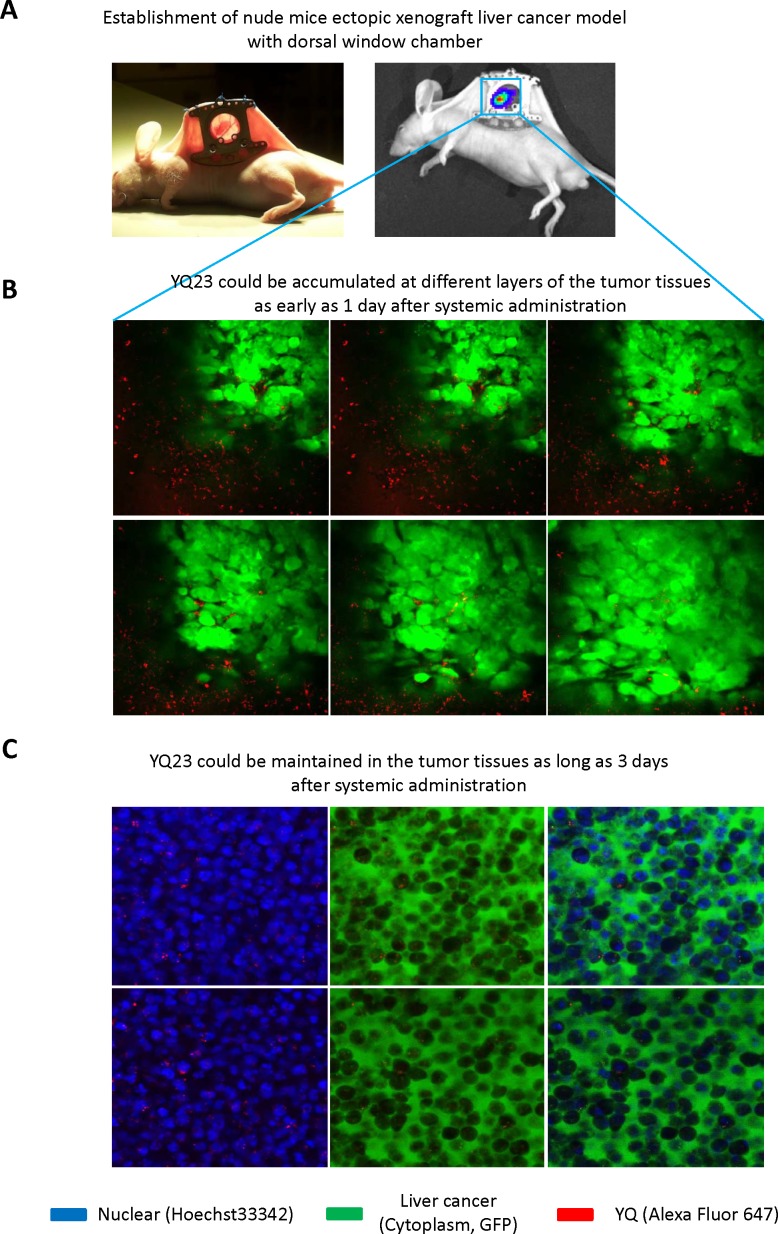
Real-time intravital imaging (Confocal) showed that YQ23 accumulated in the tumor tissue in ectopic xenograft liver cancer model using dorsal window chamber **(A)** Establishment of nude mice ectopic xenograft liver cancer model with dorsal window chamber. **(B)** YQ23 could be accumulated at different layers of the tumor tissues as early as 1 day after systemic administration. **(C)** YQ23 could be maintained in the tumor tissues as long as 3 days after administration.

We further examine whether YQ23 could be delivered into the tumor tissues using intravital imaging system. The nude mice ectopic xenograft liver cancer model was established using dorsal window chamber (Figure 6A). Through the dorsal window chamber, we can kinetically observe the drug delivery and directly see the tumor cells in a live animal. Using the real-time intravital imaging system, we observed that YQ23 could be accumulated in the tumor tissues as early as 1 day after systemic administration (Figure 6B). Moreover, we found that YQ23 could be detected in the tumor tissues as long as 3 days after administration (Figure 6C).

Intravital imagine showed that fewer YQ23 were examined within tumor tissues compared with that around the tumor tissues (Figure 6B). There may be two explanations. On one hand, the newly formed blood vessels originated from peri-tumor region. Therefore, the concentration of YQ23 was higher around the tumor tissues after systemically administration. One the other hand, the blood pressure was relatively higher within the tumor tissues due to the aggressive growth pattern of tumor cells.

### The IVIS spectrum examination showed that YQ23 distributed mainly at liver and bladder within the first 36 hours after administration in orthotopic xenograft liver cancer model

**Figure 7:**
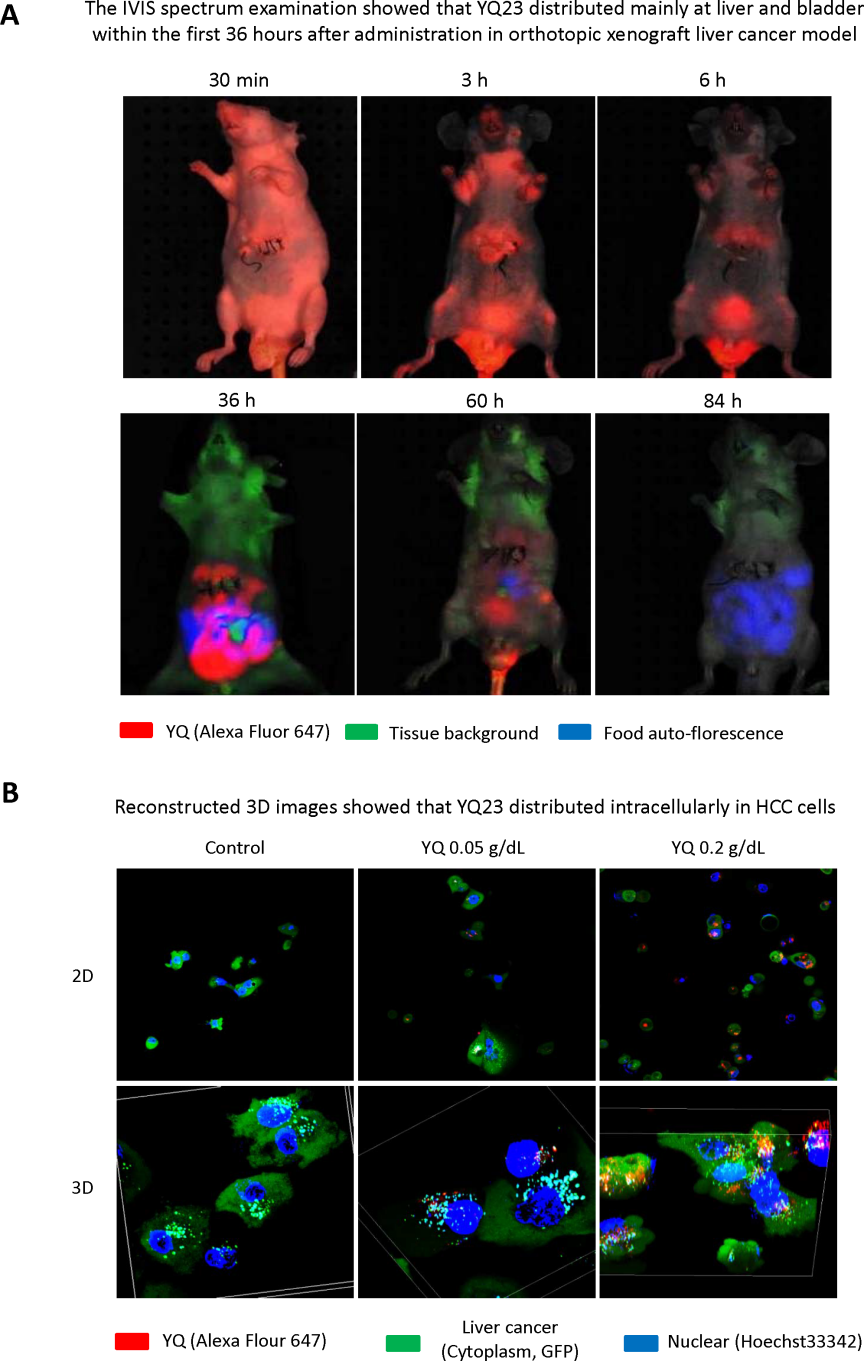
Examination of YQ distribution in the whole body and tumor cells **(A)** The IVIS spectrum examination showed that YQ23 distributed mainly at liver and bladder within first 36 hours after injection and gradually excreted through bladder afterwards. **(B)** YQ23 could be accumulated intracellularly in HCC cells as early as 1 day after administration.

In order to examine the distribution of YQ23 after injection, the nude mice orthotopic xenograft liver cancer model was established. The labeled YQ23 were injected through tail vein. The IVIS spectrum examination showed that YQ23 distributed mainly at liver and bladder within first 36 hours after injection and gradually excreted through bladder afterwards (Figure 7A).

### Reconstructed 3D images showed that YQ23 distributed intracellularly in HCC cells

In order to investigate whether YQ23 may directly penetrate into the HCC cells, the MHCC97L cells were cultured under the presence of YQ23 with different concentration. Under confocal microscope, we observed that YQ23 could be accumulated intracellularly in HCC cells as early as 1 day after administration (Figure 7B).

## DISCUSSION

In the present study, we found that the novel oxygen carrier “YQ23” significantly sensitized Cisplatin based chemotherapy in HCC cells *in vitro* and *in vivo*. Furthermore, using real-time molecular intravital system, we observed that YQ23 could be delivered into the tumor tissue, accumulated intracellularly and maintained as long as 3 days after administration. We previously reported that YQ23 can effectively improve oxygenation in liver and ameliorated hepatic IR injury after liver surgery [3]. Our recent study also showed that severe hepatic IR injury induces chemoresistance in recurrent HCC after liver transplantation [2]. Based on the present and previous studies, the oxygen carrier YQ23 may be a “one stone for two birds” therapeutic strategy not only to ameliorate hepatic IR injury, but also to sensitize Cisplatin based chemotherapy in recurrent HCC after liver transplantation.

It has been reported that hemoglobin-based oxygen carrier enhances tumor oxygenation, especially in the low oxygen tension region within tumor tissue [17]. Recently, the artificial oxygen carrier has developed as a novel therapeutic strategy for tumor oxygenation and therefore, makes the tumor cells more susceptible to current cancer treatments [17]. Systemic administration of the hemoglobin-based oxygen carrier enhances tumor oxygenation and sensitizes radiotherapy in lung cancer [18]. A recent report showed that the artificial oxygen carrier significantly promotes oxygen supply within tumor and enhances the efficacy of radiotherapy in breast cancer [19]. The sensitization of radiotherapy by oxygen carrier is mediated through induction of apoptosis in bladder cancer [20]. However, the information about the role of oxygen carrier in sensitization of chemoresistance of tumor cells is rather limited. A recent report showed that the novel oxygen carrier YQ23 significantly enhances the drug-sensitivity to 5-FU and Cisplatin treatment in esophageal squamous cell carcinoma (ESCC) [21]. Our previous study also showed that YQ23 promotes the efficacy of trans-arterial chemo-embolization (TACE) in a rat HCC model [14]. Based on our previous findings, we further explored in the present study the role of YQ23 in sensitization of chemoresistance in human HCC cells. We demonstrated, for the first time, YQ23 significantly sensitized Cisplatin-based chemotherapy in human HCC cells *in vitro* and *in vivo*. It has been proven that hemoglobin-based oxygen carrier transfusion is a safe therapeutic strategy [22, 23]. It may reduce the amount of chemotherapeutic agent in the combined treatment to achieve the similar therapeutic effect and therefore, may ameliorate the systemic side-effects in chemotherapy.

It has been established that DHFR amplification is responsible for acquired methotrexate resistance [24, 25]. Moreover, the reports showed that only a low level of amplification of DHFR gene is sufficient to induce methotrexate resistance in the patients with leukemia [26]. Not only inducing methotrexate resistance, DHFR also plays an important role in Cisplatin resistance in ovarian cancer [27]. In our study, DHFR was identified as a down-regulated gene in HCC cells upon YQ23 treatment and this finding was further validated in our animal model. This finding was also supported by the report that over-expression of DHFR is responsible for hypoxia induced chemoresistance [28]. Therefore, we provided the evidences that down-regulation of DHFR in transcription level may be one of the reasons for YQ23 sensitizing Cisplatin based chemotherapy. Investigations into the expression of translational level or protein function of DHFR in chemoresistance are worthwhile in the future study. In addition, it has been reported that hypoxia could induce Cisplatin resistance through activation of HIF-α in several types of cancer [29, 30]. However, HIF-α has not been identified as potential gene candidates in our current animal model. There might be two explanations. On one hand, the change of HIF-α expression is transient. It is difficult to be captured in animal study. On the other hand, the effect of HIF-α may be cell type specific.

The conventional measurement of delivery of oxygen carrier is examination of pO_2_ in the hypoxic tissues. Animal study for investigation of delivery of oxygen carrier always drew the conclusion based on the detection of pO_2_ at certain spots in the specific organ. However, the invasive technique is not an ideal measurement for observation of oxygen carrier delivery [31]. Hemorrhage is inevitable during detection and may influence pO_2_ dramatically [32]. Only detection at certain spots cannot indicate the distribution of oxygen carrier in the whole organ [31]. As the animal cannot tolerate anesthetization for a long time, it may avoid the possibility of kinetic observation for oxygen carrier delivery. Based on those limitations, a non-invasive technique for observation of drug delivery in a live animal is an urgent need. Currently available optical techniques of non-invasive imaging, such as MRI, PET and CT, are also limited by restricted penetration depth imposed by tissue turbidity, which avoids the possibility of utilizing high resolution microscope [15]. Real-time molecular intravital imaging with dorsal window chamber enables us to kinetically observe the delivery of oxygen carrier and directly see the tumor cells in a live animal. Utilizing the real-time intravital imaging system, we could clearly observe that YQ23 accumulated in the tumor tissue at day 1 after systemic administration and maintained as long as 3 days in ectopic xenograft liver cancer model using dorsal window chamber. Using IVIS spectrum examination, we also found that YQ23 distributed mainly at liver and bladder within the first 36 hours after administration and gradually excreted afterwards. It implied that systemic administration of YQ23 twice a week might be a practical protocol for the potential use for HCC patients in the future.

In conclusion, we demonstrated that YQ23 administration significantly suppressed the proliferation of HCC cells under Cisplatin treatment in a dose and time dependent manner. Moreover, we found that the YQ23 administration significantly sensitized Cisplatin based chemotherapy in orthotopic xenograft liver cancer model. Down-regulation of DHFR may be one of the reasons for YQ23 sensitizing Cisplatin based chemotherapy. Real-time intravital imaging showed that YQ23 accumulated in the tumor tissue and maintained as long as 3 days in ectopic xenograft liver cancer model using dorsal window chamber. The IVIS spectrum examination showed that YQ23 distributed mainly at liver and bladder within the first 36 hours and gradually excreted afterwards in orthotopic xenograft liver cancer model. We hoped that our study could provide the evidences to explore novel therapeutic strategy using oxygen carrier YQ23 to sensitize Cisplatin based thermotherapy of HCC. The role of YQ23 in amelioration of multi-drug resistance of HCC is worthwhile for further study.

## MATERIALS AND METHODS

### Cell culture

The metastatic human liver cancer cell line MHCC97L was obtained from the Liver Cancer institute and Zhongshan Hospital of Fudan University, Shanghai, the People’s Republic of China [33]. All the cell lines were cultured as previously described [34].

### Treatment regimen

YQ23 products were obtained from New B Innovation Limited [3]. YQ23 product is the stabilized non-polymeric cross-linked tetrameric hemoglobin (65 kDa) with undetectable/low level of dimeric hemoglobin (32 kDa), phospholipid, DNA impurities and protein impurities. The concentration of YQ product is 10 g/dL and its pH range is 7.4-8.4. The osmolality and viscosity (at 37°C) are 250-340 mOsm/kg and >0.9 centipoise respectively. The p50 value is ∼ 40 mmHg. The information for YQ product is shown in patent no. US7, 932, 356 B1, US 8,048,856 B1 and PCT/US12/46130. For *in vitro* study, YQ23 was administrated at final concentration of 0.0125, 0.05 and 0.2 g/dL. For *in vivo* study, YQ23 was injected through tail vein with the dosage of 0.4g/kg. For all the study, the same volume of saline was administrated in the control group. For real-time molecular intravital imaging, The YQ23 was labeled with Alex-flour-647.

### 3-(4, 5-dimethylthiazol-2-yl)-2, 5-diphenyltetrazolium bromide (MTT) assay and Colony formation assay

In order to explore the proliferation rate of HCC cells, MTT and colony formation assay were performed as previously described [34, 35].

### Animal study

Mice were housed in a standard animal laboratory with free activity and access to water and chow. They were kept under constant environment conditions with a 12 h light – dark cycle. Pentobarbitone sodium (40 mg/kg) was given intraperitoneally before any surgical procedure. Carprofen (0.1 mg in 100 ml drinking water) were used to relieve the pain for the first 3 days after operation. All animal studies were conducted according to the animal ordinance set by the government of Hong Kong. The study had been licensed according to Animal (Control of Experiments) Ordinance Chapter 340 by the Department of Health, Hong Kong Special Administrative Region. (ref.: (11–632) in DH/HA&P/8/2/3 Pt. 31).

#### Nude mice ectopic xenograft liver cancer model

MHCC97L cells (1×10^6^ cells in 100ul saline) were injected subcutaneously into right flank of the nude mice (6-8weeks, male) under anaesthesia with intraperitoneal injection of pentobarbital. When the tumor size reached 2×2mm, the dorsal window chamber was established for intravital imaging.

#### Nude mice orthotopic xenograft liver cancer model

When the subcutaneous tumors grew and reached 6mm × 6mm in size, the animals were sacrificed by intraperitoneal injection of overdose pentobarbital. The tumor tissues were harvested and cut into 1-2mm^3^ cubes. The tumor tissue cubes were then implanted into the left liver lobes of another group of mice under anaesthesia as described previously [35, 36]. Five weeks after the tumor implantation, the animals were sacrificed for samples collection and further analysis. Six mice were recruited in each group.

### RT^2^ profiler PCR array

The drug resistance related pathways were analyzed using RT^2^ Profiler PCR Array according to instruction manual (PAHS-004ZA, Qiagen). The Human Cancer Drug Resistance RT^2^ Profiler PCR Array profiles the expression of 84 genes involved in the body's response to chemotherapy. The differentially expressed gene candidates, using 2-fold as cutoff point, were identified in HCC cells (MHCC97L) after YQ (0.2g/dL) treatment for 2 days. After that, the expressions of the selected potential gene candidates were further validated in orthotopic liver cancer model.

### Real-time intravital imaging using dorsal window chamber

The dorsal window chamber (DWC) consists of two titanium frames that marry together to form a saddle on the back of the nude mouse and is attached using spacers, bolts and fastening nuts. A ‘chamber‘ is formed after a transparent glass cover slip placed onto the attached saddle covering the exposed fascia containing vessels and secured using a sterile removable ‘C’ clip. The nude mice ectopic liver cancer model was established using liver cancer cells (MHCC97L). MHCC97L cells were labeled with fluorescence reporter (GFP) during transduction and then injected subcutaneously into the window chamber from the opposing side to the glass cover-slip. The YQ23 was labeled with Alex-flour-647. The distribution of YQ23 would be kinetically observed under CZ LSM 710 confocal system in a live animal.

### IVIS spectrum examination for distribution of YQ23

In order to examine the distribution of YQ23 after injection, the nude mice orthotopic xenograft liver cancer model was established. The mice were fasted 12 hours before examination. The labeled YQ23-Alex-flour-647 was injected through tail vein. The distribution of YQ23 could be longitudinally observed by spectrum system (Perkin Elmer IVIS Spectrum). As the mice resumed food intake at later time points, the auto-florescence of food was distinguished through unmixing process.

### TUNEL assay and H&E staining

The Tunel assay and H&E staining were performed as previously described [2, 35, 37, 38].

### Statistical analysis

The Chi-square test was used to compare categorical data. Paired or unpaired T test were adopted to compare continuous variables. *P* < 0.05 was considered as statistically significant. Calculation was made using SPSS computer software version 16 (SPSS Inc, Chicago, IL, USA).

## SUPPLEMENTARY MATERIALS FIGURE



## Abbreviations

IR: ischemia-reperfusion
HCC: hepatocellular carcinoma
qRT-RCR: real time quantitative reverse transcription polymerize chain reaction
IHC: immunohistochemistry
HE: hematoxylin and eosin
MTT: 3 - (4, 5 – dimethylthiazol – 2 - yl) - 2, 5 - diphenyltetrazolium bromide
DWC: The dorsal window chamber
DHFR: dihydrofolate reductase
