# Supplementary material for: A hemoglobin-based oxygen carrier sensitized Cisplatin based chemotherapy in hepatocellular carcinoma
Source: Oncotarget. 2017 Jul 28;8(49):85311–25. doi: 10.18632/oncotarget.19672 (PMC5689611; doi:10.18632/oncotarget.19672)
Supplement: Supplementary file 1 [file oncotarget-08-85311-s001.pdf]

A hemoglobin-based oxygen carrier sensitized Cisplatin based chemotherapy in hepatocellular carcinoma

SUPPLEMENTARY MATERIALS

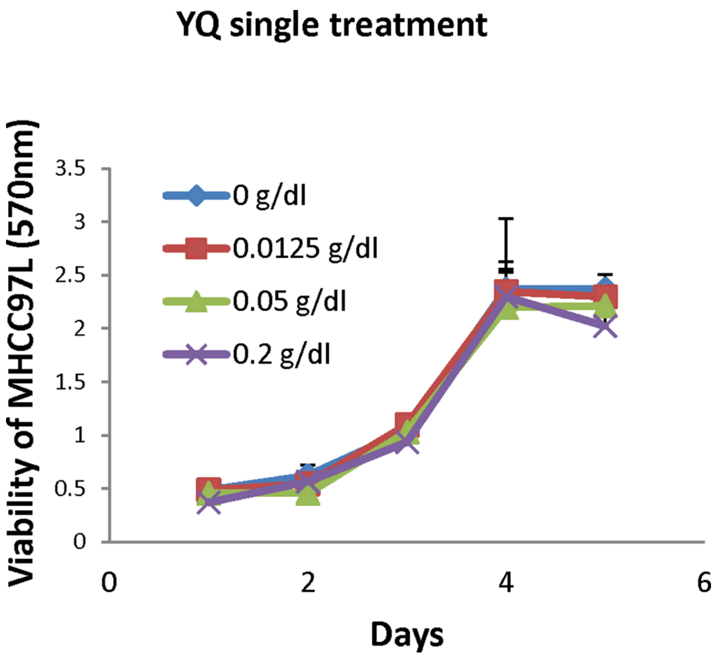

Supplementary Figure 1: No significant effect of single treatment of YQ23 was observed in HCC cells.
